# Patterns of Genome-Wide Variation in *Glossina fuscipes fuscipes* Tsetse Flies from Uganda

**DOI:** 10.1534/g3.116.027235

**Published:** 2016-03-26

**Authors:** Andrea Gloria-Soria, W. Augustine Dunn, Erich L. Telleria, Benjamin R. Evans, Loyce Okedi, Richard Echodu, Wesley C. Warren, Michael J. Montague, Serap Aksoy, Adalgisa Caccone

**Affiliations:** * Department of Ecology and Evolutionary Biology, Yale University, New Haven, Connecticut 06511; † Laboratorio de Fisiologia e Controle de Artropodes Vetores, Instituto Oswaldo Cruz, Fiocruz, Rio de Janeiro, 21040-360 RJ, Brazil; ‡ Department of Biology, Faculty of Science, Gulu University, Loroo Division, 256 Gulu, Uganda; § The Genome Institute, McDonnell Genome Institute, Washington University School of Medicine, St Louis, Missouri 63108; ** School of Public Health, Yale University, New Haven, Connecticut 06510

**Keywords:** tsetse flies, linkage disequilibrium, association studies, population genomics, ddRAD

## Abstract

The tsetse fly *Glossina fuscipes fuscipes* (*Gff*) is the insect vector of the two forms of Human African Trypanosomiasis (HAT) that exist in Uganda. Understanding *Gff* population dynamics, and the underlying genetics of epidemiologically relevant phenotypes is key to reducing disease transmission. Using ddRAD sequence technology, complemented with whole-genome sequencing, we developed a panel of ∼73,000 single-nucleotide polymorphisms (SNPs) distributed across the *Gff* genome that can be used for population genomics and to perform genome-wide-association studies. We used these markers to estimate genomic patterns of linkage disequilibrium (LD) in *Gff*, and used the information, in combination with outlier-locus detection tests, to identify candidate regions of the genome under selection. LD in individual populations decays to half of its maximum value (*r*^2^_max_/2) between 1359 and 2429 bp. The overall LD estimated for the species reaches *r*^2^_max_/2 at 708 bp, an order of magnitude slower than in *Drosophila*. Using 53 infected (*Trypanosoma* spp.) and uninfected flies from four genetically distinct Ugandan populations adapted to different environmental conditions, we were able to identify SNPs associated with the infection status of the fly and local environmental adaptation. The extent of LD in *Gff* likely facilitated the detection of loci under selection, despite the small sample size. Furthermore, it is probable that LD in the regions identified is much higher than the average genomic LD due to strong selection. Our results show that even modest sample sizes can reveal significant genetic associations in this species, which has implications for future studies given the difficulties of collecting field specimens with contrasting phenotypes for association analysis.

African trypanosomiasis negatively impacts both human and animal health in sub-Saharan Africa (Simarro *et al.* 2012a). In 2008, mortality associated with Human African Trypanosomiasis (HAT or sleeping sickness) ranked ninth out of 25 human infectious and parasitic diseases in Africa (Fevre *et al.* 2008). The causative agents of HAT are members of the genus *Trypanosoma* (*Kinetoplastida*), species *T. brucei rhodesiense* (*Tbr*) and *T. b. gambiense* (*Tbg*), while Animal African Trypanosomiasis (AAT or Nagana) is caused by *T. b. brucei* (*Tbb*), *T. congolense*, and *T. vivax*. HAT caused by *Tbg* is a chronic disease with asymptomatic periods lasting several years, while *Tbr* infection results in an acute disease with over 80% mortality within the first 6 months if untreated. Over 90% of HAT cases are due to *Tbg*, which occurs in the northwest regions of Uganda and extends from Central African Republic to Equatorial Guinea. More than 12 million people in eastern and southern Africa, including Uganda, Tanzania, Malawi, Zambia, and Zimbabwe, are at risk for *Tbr* infection (Simarro *et al.* 2012a). Intense international interventions have reduced HAT cases to below 10,000 for the first time in 50 yr (Simarro *et al.* 2011), but many cases likely go undetected in remote regions (Aksoy 2011; Simarro *et al.* 2011). Since available drugs for treatment are expensive, associated with adverse effects (Simarro *et al.* 2012b), and exhibit reduced efficacy due to increasing drug resistance in the parasites (Brun *et al.* 2001), the most effective current control methods involve reduction of the vector populations: tsetse flies that belong to the genus *Glossina*.

Uganda is the only African country where both forms of HAT exist, with *Tbg* occurring in the northwest and *Tbr* in the southeast areas (Figure 1). The two forms of the disease are feared to merge in the near future, as the belt that currently separates them is less than 100 km wide (Picozzi *et al.* 2005). Given that the pathology, diagnosis, and treatment of HAT for *Tbr* and *Tbg* disease vary significantly, the overlap of the two disease belts is expected to complicate HAT control (Welburn *et al.* 2009). *Glossina fuscipes fuscipes* (*Gff*) is the vector for both forms of HAT in Uganda. To understand the biological processes behind disease spread, especially in light of the impending disease merger, we had previously performed population genetics studies on Ugandan populations of *Gff* and identified three genetically and geographically distinct population clusters (northern, western, and southern Uganda), based on 12–15 microsatellite loci (Hyseni *et al.* 2012; Echodu *et al.* 2013; and Figure 1). These genetic clusters display high intercluster *F*_st_ values (0.124–0.574), typically associated with insects that differ at the subspecies level, or with the possibility of coexistence of distinct species (Beadell *et al.* 2010; Hyseni *et al.* 2012). The northern and southern clusters of *Gff* meet in a narrow hybrid zone in central Uganda along Lake Kyoga (Beadell *et al.* 2010). Our results have also shown that *Gff* populations are genetically stable over time (Beadell *et al.* 2010; Echodu *et al.* 2011; Hyseni *et al.* 2012), that genetic admixing occurs among sites from the same or different clusters within a 100 km radius (Beadell *et al.* 2010), and that gene flow is not symmetrical, being three times greater from the southeast to northwest than in other directions (Hyseni *et al.* 2012). Given that *Gff* is a riverine species that survives in habitats with high humidity (Hargrove 2001a, 2001b; Terblanche *et al.* 2008; Nash 1933; Rogers 1979), the asymmetric gene flow could be tied to the influence of the Nile River and its tributaries that flow from Lake Victoria in the south through Lake Kyoga to Lake Albert in northern Uganda (Beadell *et al.* 2010).

**Figure 1:**
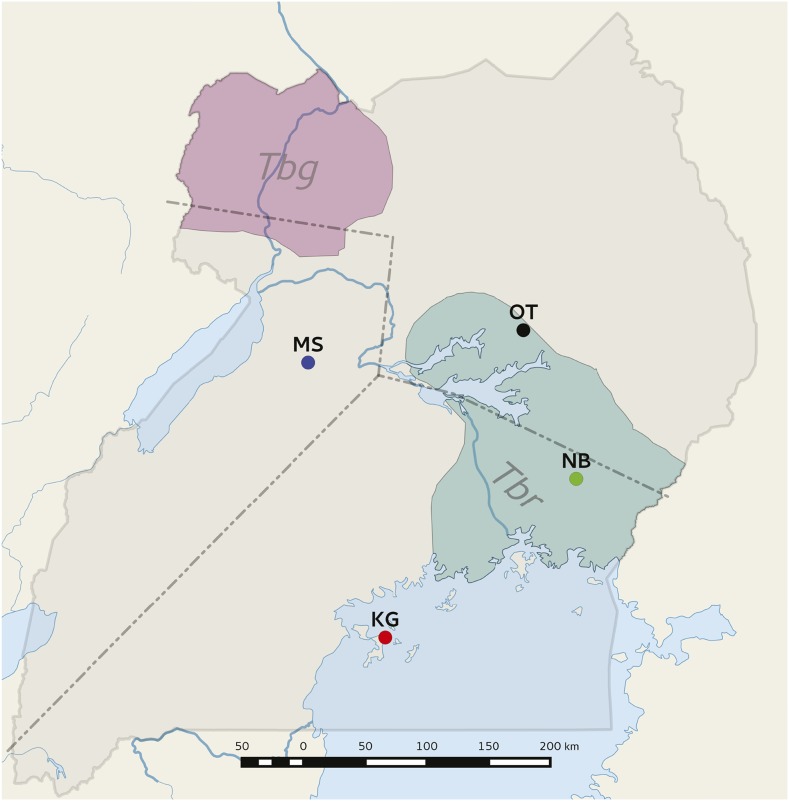
Geographical distribution of *Glossina fuscipes fuscipes* collections sites in Uganda. Collections from Masindi (MS), Kalangala Island (KG), Otuboi (OT), and Namutumba (NB) were used in this study. Broken lines mark the hypothetical separation of the three main genetic clusters based on microsatellite data (Hyseni *et al.* 2012; Echodu *et al.* 2013). Red and green shading indicates the distribution range of *Trypanosoma brucei gambiense* (*Tbg*) and *T.b. rhodesiense* (*Tbr*).

Although traditional population genetic markers such as mitochondrial and microsatellite loci have provided important insights into the population dynamics of *Gff*, genome-wide approaches that rely on thousands of polymorphic markers are required to further investigate the patterns of neutral and adaptive genetic variation in wild populations. Genome scans of single copy nucleotide polymorphisms (SNPs) using next-generation DNA sequencing (NGS) can be used to detect signatures of adaptive genetic variation in wild populations without *a priori* identification of candidate genes (Elmer and Meyer 2011). These methods allow simultaneous screening of thousands of genetic markers, and large numbers of individuals (Storz 2005; Ekblom and Galindo 2011; Seeb *et al.* 2011; Narum *et al.* 2013; reviewed by Glenn 2011), and are often coupled with sampling designs and statistical methods that take into account drift and geographic differentiation (Stinchcombe and Hoekstra 2008; Storz 2005; Storz and Wheat 2010; Jones *et al.* 2012). This approach can be used for both model and nonmodel organisms, regardless of the availability of a reference genome (Bonin *et al.* 2006; Egan *et al.* 2008; Hohenlohe *et al.* 2010; Stapley *et al.* 2010; Anderson *et al.* 2012; Nosil and Feder 2012; Orsini *et al.* 2012).

Here, we used double digestion restriction associated RADSeq (Peterson *et al.* 2012) in combination with whole genome sequencing (WGS) to develop a SNP toolbox for studying *Gff* populations in Uganda. We carried out this work using *Gff* flies from four sampling sites, representative of the three previously described genetic clusters (two from the southern genetic cluster, and one each from the other two clusters; Echodu *et al.* 2013). The inclusion of multiple genetic populations, which maximizes the representation of the genetic diversity of the Ugandan *Gff* populations, aims to reduce false positives when looking for adaptive variation by minimizing ascertainment bias (reviewed by Schoville *et al.* 2012). Since adaptive processes are restricted to specific genes/gene regions, while drift and gene flow would affect all genes equally, the analysis of different genetic backgrounds facilitates the distinction of forces shaping the distribution of neutral and adaptive genetic variation, and thus should help to identify the genetic underpinnings of specific traits (Luikart *et al.* 2003).

While our main goal was to identify SNPs for future population genomic studies, this dataset provided us with the opportunity to estimate, for the first time, *Gff* genomic patterns of LD, and to compare the estimates of genetic differentiation from the SNP data with those from previous studies with microsatellite and mtDNA markers. We also used these data to look for associations between SNPs and two epidemiologically relevant traits: susceptibility to *Trypanosoma* infections, and environmental adaptation. We discuss the epidemiological relevance of this work, and the future research avenues it enables.

## Materials and Methods

### Glossina fuscipes fuscipes (Gff) samples

*Glossina f. fuscipes* samples were collected from four sites in Uganda in 2010 and 2011 (Table 1, Figure 1, and Supplemental Material, Table S1): Masindi (MS), Otuboi (OT), Namutumba (NB), and Kalangala Island (KG). Flies from each site included in this study had been previously analyzed using microsatellite and/or mtDNA loci (Beadell *et al.* 2010; Echodu *et al.* 2011, 2013; Hyseni *et al.* 2012). Based on these markers, they belong to three distinct genetic clusters located at the north, south, and north-west of Lake Kyoga (Figure 1). Collections were carried out using biconical traps, and each fly was stored individually in 80% ethanol until DNA extraction. Table 1 lists the number of samples per site, the year of collection, and their *Trypanosoma* infection status; details for each individual sample are provided in Table S1. All individuals included in this study were adults, but their specific age could not be determined. All samples were collected in 2011, except for the individuals from Kalangala, which were collected in 2010. However, given that the results from previous studies suggest that allelic frequencies are stable over time in *Gff* populations in Uganda (Echodu *et al.* 2013), we included this population in the analyses, unless stated otherwise.

**Table 1:** Sample summary

Sequencing Method	No. of Populations	No. of Individuals	Population Name	Year Collected	Symbol	Infected/ Uninfected
Whole-genome Sequencing	1	16	Namutumba	2011	NB	8/8*^a^*
ddRAD sequencing	4	48	Masindi	2011	MS	7/7
			Otuboi	2011	OT	7/7
			Namutumba	2011	NB	8/8
			Kalangala	2010	KG	2/2

a Six of these samples were also included in the ddRAD sequencing set (5/1).

### DNA extraction and assessment of Trypanosoma infection by PCR

DNA was extracted from carcasses and midguts using the MasterPure Complete DNA Purification Kit (Epicentre Biotechnologies), following the manufacturer’s protocol.

DNA from legs was extracted using the DNeasy Blood and Tissue kit (Qiagen) according to the manufacturer’s instructions. All extractions were stored at –20° until further processing. Each sample was tested for *Trypanosoma* infection twice via PCR amplification using trypanosome alpha-tubulin primers (trypalphatubF: CTCGACACACTCACTTCTGGAG; trypalphatubR: CGAATTTGTGGTCAATACGAG). This assay was not specific for trypanosomes that are human infective (*i.e.*, *Tbr* or *Tbg*), but rather detects the presence of any *Trypanosoma* species, including *T. brucei*, *T. congolense*, and *T. vivax*. Table 1 reports the number of infected and uninfected flies for each sampling site.

### Reduced representation genomic libraries

#### ddRADseq:

Double-digest restriction site-associated DNA sequencing libraries (ddRAD) were prepared using a modified version of the protocol described in Peterson *et al.* (2012). Briefly, ∼1 μg of genomic DNA from each *Gff* individual was digested with restriction enzymes *Nla*III and *Mlu*CI (NEB). Each individual sample was then labeled with custom Illumina adaptors carrying a unique barcode at the 5′ end of the digested fragments. The fragments were amplified by eight cycles of polymerase chain reaction (PCR) to select for those fragments containing different restriction sites at each end, and increase the total concentration of the desired fragments. After PCR amplification, a library was constructed by pooling the products of 24 individuals and later size-selecting them to a fragment size of 215 bp under the “tight” setting of a Blue Pippin electrophoresis platform (Sage Science). Libraries were sent to the Yale Center for Genome Analysis for 75 bp paired-read sequencing with the Illumina Hi-Seq platform. To achieve the best sequence quality, the complexity of the sequencing lanes was increased by spiking the libraries with a secondary library whose fragments did not begin with the restriction sites used by our ddRAD library. Data are available via BioSample project accession number PRJNA303153, with linked associated short-read sequences and variation data.

### Whole-genome-sequencing

Whole genomes of 16 individuals from the NB population were sequenced at the Genome Institute of the Washington University School of Medicine (St Louis, MO) as 100-bp reads using the Illumina HiSeq2000 platform at ∼40 × coverage (Table 1 and Table S1). All sequence data for each of the 16 samples was trimmed using flexbar version 2.4 (Dodt *et al.* 2012) prior to alignment. Synonymous site identification to perform genetic diversity calculations was performed using the GfusI1.1 gene-build (VectorBase 2014; Giraldo-Calderon *et al.* 2015) with the variant effect predictor (VEP) tool within Ensembl (Flicek *et al.* 2014) to determine the genomic context and potential function of all identified variants. The software annotates all SNPs as belonging to one of several functional categories: stop-gained, stop-lost, frameshift-coding, nonsynonymous-coding, splice-site, promoter, 5′ UTR, 3′ UTR, upstream, downstream, intronic; synonymous coding or intergenic. Subsequently, only variants with synonymous effects were parsed for each sample in order to compile a list of synonymous variant sites across the genome assembly. Genetic diversity (π), and Tajima’s *D* values (5000-bp nonoverlapping windows) were calculated using vcftools v. 0.1.12b (Danecek *et al.* 2011) and average values obtained within the R software (R Core Team 2013).

### Data processing

The ddRAD library raw sequence reads were demultiplexed, quality filtered, and filtered for unambiguous barcodes using “*process_radtags*” from the Stacks software (Catchen *et al.* 2011). This dataset was then used to call SNPs using the *G. fuscipes* reference assembly as described in the next section. To improve the coverage of individuals for which not enough reads were obtained by the original ddRAD run due to the low quality of the sample, we combined the reads with the data from WGS of 16 individuals from the NB population. Combined, our dataset included 58 individuals, as six of them were sequenced with both technologies (Table 1 and Table S1). The software PGDSpider v. 2.0.5.2 (Lischer and Excoffier 2012) was used to convert between file formats for downstream analyses.

### Mapping and variant calling and summary statistics

Polymorphic loci were identified from the combined reads of the 58 individuals (ddRAD and WGS) by mapping them against the 2395 supercontigs of the *G. fuscipes* GfusI1 reference assembly (VectorBase 2014; Giraldo-Calderon *et al.* 2015) using Bowtie2 v. 2.1.0 (Langmead and Salzberg 2012) in the “very sensitive” option, and Samtools v. 0.1.19 (Li *et al.* 2009). Variants were called using the bcftools utility from Samtools, and data filtered in vcftools v. 0.1.10 (Danecek *et al.* 2011) based on genotype depth of coverage (DP > 7) and percentage of missing data allowed (< 30%). Only loci that genotyped in at least 80% of the samples were included in subsequent analysis. Our final variant calling file (vcf format) contained only biallelic SNPs and no indels. Five individuals that did not genotype for at least 80% of SNPs were removed from the analysis, and a second filtering was performed on the remaining 53 individuals, including a minor allele frequency filter (MAF > 0.05). All summary statistics including depth of coverage, SNP density, Hardy-Weinberg equilibrium tests, Tajima’s *D*, and between populations *F*_st_, were performed using vcftools v. 0.1.12b (Danecek *et al.* 2011), and processed with the R software (R Core Team 2013). Tajima’s *D* values were estimated using a nonoverlapping window size of 1000 bp. Tajima’s *D* values are indicative of the presence and direction of selection in the region. In general, values above 2 are considered significant, with positive values suggesting balancing selection, and negative values the presence of a selective sweep or a bottleneck (Anholt and Mackay 2010).

### Cluster analyses

Two different clustering methods were used to determine the presence of underlying population structure in our samples. First, we performed a Bayesian clustering analysis in fastStructure (Raj *et al.* 2014), using only loci in Hardy-Weinberg equilibrium (HWE) after Benjamini-Hochberg (BH) correction (*P* ≤ 0.05) (Benjamini and Hochberg 1995). fastStructure uses variational Bayesian inference, and large numbers of SNPs to assign individuals probabilistically to *K* numbers of clusters characterized by a set of allele frequencies at each loci, with no *a priori* information of sample location. This method assumes HWE and no linkage disequilibrium (LD) among loci. In addition to detecting existing population structure, fastStructure provides ancestry estimates of each of the sampled individuals. Ten independent fastStructure runs were conducted with *K* = 1–5 on all individuals of all populations. The optimal number of *K* clusters was then determined using the *chooseK.py* program of fastStructure. Results were plotted with the program DISTRUCT v.1.1 (Rosenberg 2004). Second, we conducted principal component analysis (PCA) of the entire dataset using the Adegenet package v. 1.3.9. (Jombart 2008) available for the R software v. 3.0.1. (R Core Team 2013). PCA provides a simple, low-dimensional, projection of the data by performing single value transformation on multiple possibly correlated variables, allowing for visual identification of existing population structure.

### Linkage analysis

#### Linkage measurements:

Vcftools version 0.1.12 (Danecek *et al.* 2011) was used to calculate pairwise linkage disequilibrium (LD) as *r*^2^ (Hill and Robertson 1968) for all SNP-pairs located on common supercontigs. The “–allow-extra-chr” option was required to handle the number of supercontigs (*N* = 2395). The KG population was omitted from the analysis due to its low sample size. Unless stated otherwise, all subsequent analysis pertaining to LD used *r*^2^.

LD values of all SNP-pairs were compared after binning SNP-pairs by physical distance (bp) to control for unknown rates of recombination in *Gff*. Bin length was set at 50 bp. To identify SNP-pairs with abnormal LD values, we assigned probabilities to each SNP-pair in the bin. As the distributions of binned SNP-pairs are bounded by 0 and 1 and do not appear to be Normal in shape, and because in many cases the data appear to exhibit peaks at both the lower and upper *r*^2^ range, the data were modeled using the probability density function (PDF) of the Beta distribution. A PDF describes the relative likelihood that a random variable will take a particular value, or range of values, given the type of probability distribution. Using the cumulative distribution function (CDF) of the Beta distribution, we can describe the probability that a binned SNP-pair will have an *r*^2^ less than or equal to *x*. It follows that 1 − CDF represents the probability of observing a more extreme value than a particular *r*^2^ value. For each set of binned *r*^2^ values, the SNP-pairs deemed worthy of further investigation were defined as those where 1 − CDF ≤ 0.01 after BH correction for multiple testing.

The Beta distribution is bounded on the noninclusive interval between 0 and 1, meaning no value is expected to equal *exactly* 0 or 1. However, there are data in each bin that have been assigned values of 0 or 1. It is likely that these values are not truly 0 or 1 in the discrete binary sense that a coin-flip can produce *only* a heads or tails result. Therefore, to satisfy the expectations of the Beta distribution, all *r^2^* values for each bin were scaled according to the scheme:

$$\left(\left(x_{\text{i}}-0.5\right)\cdot \text{θ}\right)+0.5$$

in order to symmetrically shrink the distribution of *r*^2^ values to fit between 0 and 1, while not including any values equal to 0 or 1. In the scheme above, let *x*_i_ stand for the *r*^2^ of each SNP-pair in an arbitrary bin, and θ stand for the scaling factor. The results in this paper used θ = 0.999.

### Selection analysis

As we hypothesize that SNP variation is associated with phenotypic and environmental variation, we carried out genome scans to identify possible genomic regions under selection associated with the infection status of the fly, assessed via PCR (*Trypanosoma* infected *vs.* uninfected), or with local environmental adaptation, determined by the PCA loadings of the environmental parameters corresponding to each geographic location obtained from the WorldClim database (Hijmans *et al.* 2005; File S1).

We used multiple complementary methods to detect signatures of adaptive change while accounting for drift, geographic structure, and a small sample size. At the individual loci level, we used BayeScan (Fischer *et al.* 2011), known to perform well with very small sample sizes; and PCAdapt (Duforet-Frebourg *et al.* 2014), which accounts for population structure and specifically identifies loci associated with environmental adaptation. We also used a haplotype-based approach implemented by hapFLK, (Fariello *et al.* 2013), which incorporates the hierarchical structure of the populations in the analysis, is robust to the demographic history of the populations, and is capable of detecting incomplete selective sweeps that are difficult to detect with methods that rely on allele frequencies. Details of the selection screens can be found in File S2. Furthermore, we use our estimates of LD to further analyze the BayeScan results to identify loci that might be under selection, but were not recognized as outliers due to insufficient statistical power. This was achieved by applying an LD filter to the dataset of SNPs ranked in BayeScan within the top 10% alpha values. Those SNPs that were part of an SNP-pair with an assigned probability value of *P* ≤ 0.01, based on a Beta probability distribution, were considered potential candidate loci under selection (see previous section).

### Functional annotations and identification of candidate genes

The SNPs identified by each of the above methods were then mapped to the annotated *Gff* genome and transcriptome to identify potentially relevant genes in the proximity. We searched for genomic regions under selection on individuals from the NB, MS, and OT populations. Population KG was excluded from the analysis due to the low number of individuals sampled.

All putative peptides annotated for *Gff* in the GfusI1.1 gene-build were obtained from VectorBase (2014); Giraldo-Calderon *et al.* 2015. The sequences used to compare the *Gff* peptides against well known/annotated sequences were obtained from UniProt/SwissProt (Boeckmann *et al.* 2005), used with blastp, and Pfam (Finn *et al.* 2014), used with hmmscan, as required by ARGOT2 (Falda *et al.* 2012; Gillis and Pavlidis 2013; Radivojac *et al.* 2013). The blastp and hmmscan results submitted to ARGOT2 were obtained by performing local searches on the *Gff* peptides against the UniProt peptide database (obtained on September 8, 2014), and the hidden Markov models (HMM) of the combined protein-domain sets on the Pfam databases (Pfam-A and Pfam-B: obtained on September 8, 2014), respectively. Settings used were as dictated by the ARGOT2 site. The blastp and hmmscan results were uploaded to ARGOT2 servers for analysis after being split into 10 groups (∼2330 peptides per group) to prevent overloading the remote ARGOT2 cluster. The functional annotations were then downloaded and joined back together.

### Data availability

The Supplementary Material files contain supplementary Figure S1, Figure S2, Figure S3, Figure S4, Figure S5, and Figure S6, and their legends, as well as Table S4 and the legends of all supplementary tables and supplementary files. File S1 contains the data and analysis of the bioclimatic parameters. File S2 contains details of the selection analyses. File S3 lists the genes located within 1000 bp of SNPs identified by the selection analysis in BayeScan, associated to local environmental adaptation and susceptibility to trypanosome infection. File S4 lists the SNPs identified as outliers in BayeScan during the pairwise population comparisons. The *Gff* Illumina read sequences produced in this study are available as part of BioSample project accession number PRJNA303153 with linked variation data (ddRAD), and project accession number SAMN02742630 (WGS). Data were deposited at BioSample project under accession numbers PRJNA303153 and SAMN02742630.

## Results

### Marker discovery

Approximately 15% of the fragments generated after *in vitro* digestion of *Gff* genomic DNA using the restriction enzymes *Nla*III and *Mlu*CI were 172 to 216 bp in length (193 bp on average), the size chosen for constructing the ddRAD libraries. Downstream analysis of the generated ddRAD reads indicates that we achieved an average coverage of 32 × for each individual fly.

We recovered a total of 448,881,370 reads from the 48 individuals subjected to ddRAD sequencing. Quality processing of these reads with the software Stacks (Catchen *et al.* 2011) yielded a total of 428,673,526 high quality reads (95%) with unambiguous barcodes. Combining the ddRAD reads for the NB population with the WGS reads (see *Materials and Methods*) allowed us to increase the coverage of individuals of this population that were underrepresented in the ddRAD libraries due to low quality samples. With this strategy we recovered 37 × more SNPs for the NB population than those obtained from the ddRAD seq alone, after the appropriate filtering was performed. The dataset containing the filtered ddRAD reads for all four Ugandan populations, and the filtered WGS reads for the NB population was subsequently used to call variant sites.

The *Gff* GfusI1 reference assembly (VectorBase 2014; Giraldo-Calderon *et al.* 2015) used for variant calling extends ∼375 Mbp, broken down into 2395 supercontigs that range in size from 886 bp to 3,329,503 bp (mean = 156,482 bp, and median = 19,838 bp). A total of 5,246,046 SNPs were obtained after mapping the combined dataset to this reference (see *Materials and Methods*). Further filtering for biallelic SNPs, with a minimum depth of coverage of 7 ×, and present in at least 80% of the samples, resulted in a set of 153,663 SNPs. Individuals KG10_030, MS11_0017, MS11_0050, NB11_056, and NB11_062 were removed from subsequent analyses because they genotyped for < 80% of the SNPs called. This dataset, containing 53 individual flies, was filtered once more to remove any sites that were polymorphic only in the individuals removed. The final set included 73,297 SNPs, with an average coverage of 41.89 × ± 17.46 × per site, ranging from 18.76 × to 102.78 × (median = 40.31 ×). The final dataset was used for the downstream analyses described below.

We identified SNPs in 1389 of the assembled *Gff* supercontigs (58% of the total), with an average of two SNPs for every 10,000 bp. The number of SNPs per supercontig ranged from zero to 25 at the 10,000 bp window size (median = 1 SNP / 10,000 bp). Analysis using a window size of 1000 bp yielded an average of 0.2 SNPs / 1000 bp, with some supercontigs having no SNPs, and 26 contigs having 10 or more.

### Genetic diversity and differentiation

Observed individual heterozygosities (*H*_o_) ranged from 0.1111 to 0.3435 (mean *H*_o_ = 0.2156 ± 0.0477). Average heterozygosities per population were *H*_oKG_ = 0.4631, *H*_oOT_ = 0.3404, *H*_oNB_ = 0.3203, and *H*_oMS_ = 0.2968. Average Tajima’s *D* across all populations and considering 1000 bp nonoverlapping windows (regardless of whether they contain SNPs or not) was 0.0526. Mean Tajima’s *D* values considering only those 1000 bp windows that contain at least one SNP was 0.4055, with absolute values larger than |2| detected in 2684 of the windows, and 77 of them displaying values larger than |3| (Table S2 and Figure S1).

Estimates of genome-wide genetic diversity calculated from the WGS data available for the NB population were π = 0.00056, with an average Tajima’s *D* of –0.0782. When only synonymous sites were considered, these values were π = 0. 0.0001, with an average Tajima’s *D* of 0.0828.

Values of between-population genetic differentiation (*F*_st_) are shown in Table 2. Genetic differentiation was higher between KG and OT (*F*_st_ = 0.3214); and between NB and OT (*F*_st_ = 0.3015). The lowest degree of differentiation was found between KG and NB (*F*_st_ = 0.0853). The average pairwise *F*_st_ = 0.2877 (weighted *F*_st_ = 0.3409).

**Table 2:** Pairwise *F*_st_ values between populations of *Glossina fuscipes fuscipes* from Uganda

	NB	OT	MS	KG
NB	0	0.42626	0.21029	0.18811
OT	0.30145	0	0.34275	0.47684
MS	0.16772	0.24805	0	0.26597
KG	0.08532	0.32144	0.14583	0

*F*_st_ values are above the diagonal; weighted *F*_st_ values are below the diagonal. Weighted *F*_st_ is calculated in vcftools v. 0.1.12b (Danecek *et al.* 2011), using the Weir and Cockerham (1984) estimator to correct for samples size.

We identified 6180 loci that deviated from HWE after Bonferroni (Bf) correction (*P*_Bf_ ≤ 0.05), and 25,300 loci that deviated from HWE when BH correction was applied instead (*P*_BH_ ≤ 0.05). These latter loci were not considered for the Bayesian clustering analysis in fastStructure, which assumes HWE (see next section). When populations were analyzed individually, no loci deviated from HWE in KG, MS, and OT after either Bf or BH correction was applied. However, in the NB population, 278 (Bf) or 936 (BH) loci were out of HWE.

**Figure 2:**
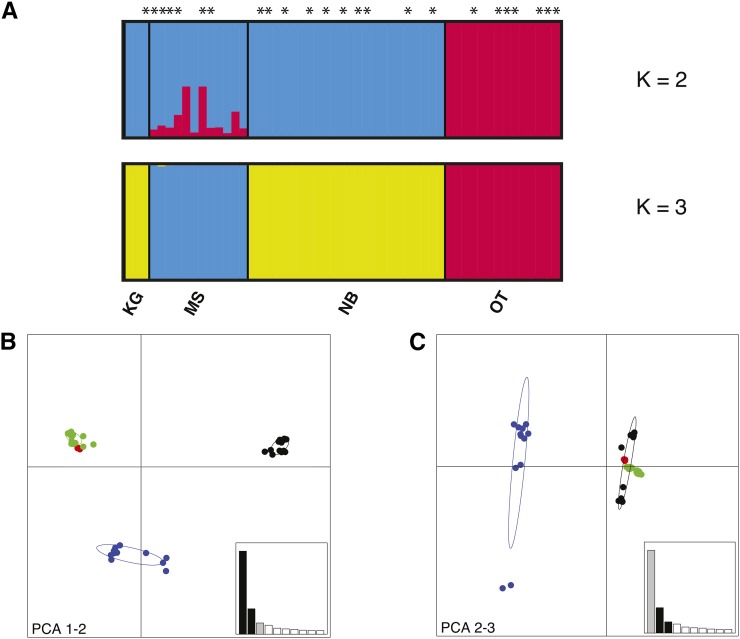
Population structure in *Glossina fuscipes fuscipes* (*Gff*) from Uganda. (A) Genetic membership bar plot based on the 47,997 SNPs that met HWE expectations after BH correction obtained using fastStructure (Raj *et al.* 2014). Each vertical bar represents a single individual. The height of each color represents the probability of assignment to each of *K* clusters. Individuals infected with *Trypanosoma* are indicated with and asterisk (*). Plots of *K* = 2 and *K* = 3 are shown for all sampled flies in all four populations (*N* = 53). (B)–(C) Principal component analysis (PCA) plots of *Gff* populations based on all 73,297 SNPs. Each dot represents an individual. The 95% inertia ellipses are shown for each population. (B) PC1 *vs.* PC2. (C) PC2 *vs.* PC3. Locations are identified by different colors. Variance explained by the different components = PCA1: 0.3%, PCA 2: 0.09%, PCA3: 0.04%. KG, Kalangala Island (Red); MS, Masindi (Blue); OT, Otuboi (Black); and NB, Namutumba (Green).

Bayesian clustering analysis in fastStructure (Raj *et al.* 2014) suggests *K* = 3 as the most likely number of genetic groups, with KG clustering together with NB (Figure 2A). Clustering is based on geography rather than infection status at both *K* = 2 and *K* = 3 (Figure 2A). Although this analysis was run using only loci in HWE to meet the assumptions of the test, the pattern remained unchanged when all 73,297 loci of the dataset were included (Figure S2). PCA of the data shows the same pattern as the Bayesian clustering (Figure 2B), with the KG population grouping with NB. PC1 separates these two populations from OT and MS. OT is placed with KG and NB by PC3, leaving MS as an independent cluster (Figure 2B).

### Linkage disequilibrium

**Figure 3:**
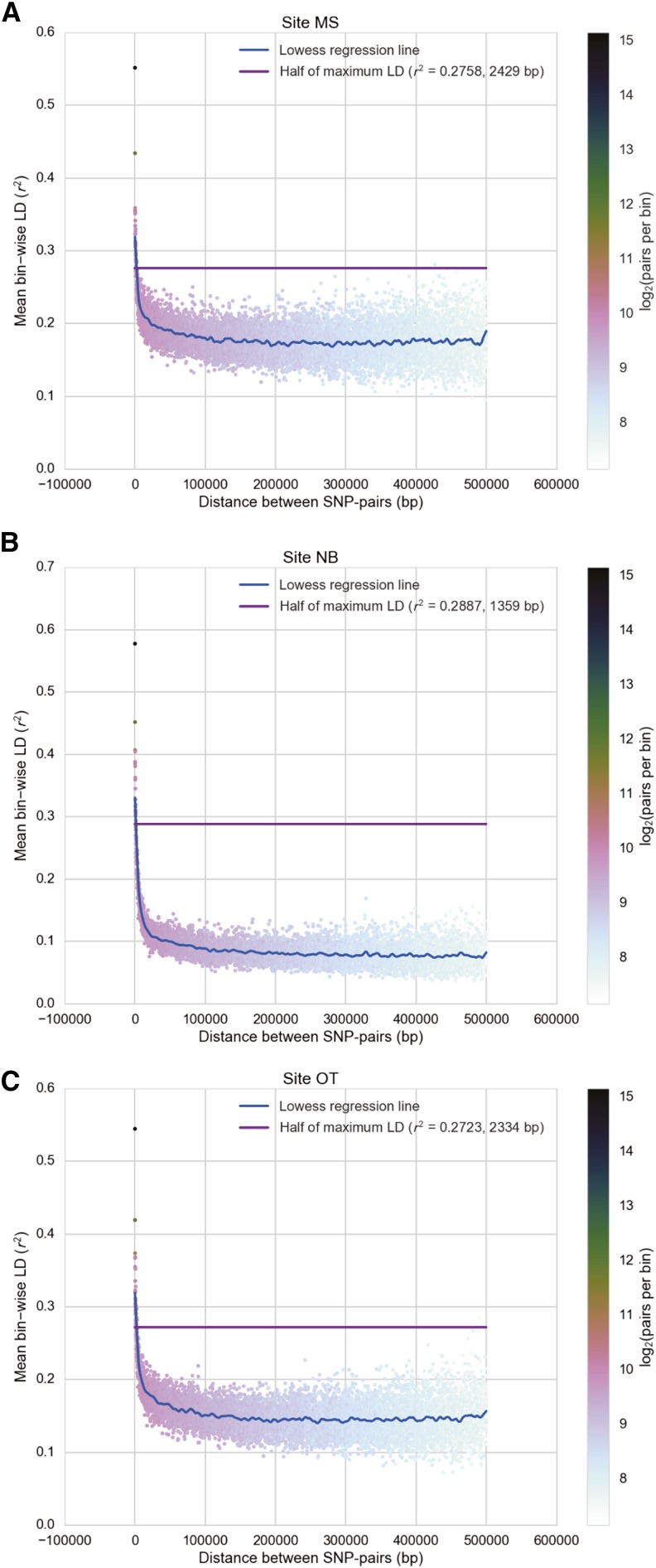
The rate of linkage disequilibrium (LD) varies with the *Glossina fuscipes fuscipes* population. Pairwise LD between SNPs located in the same supercontig was estimated from all individuals of all populations using Vcftools v. 0.1.12 (Danecek *et al.* 2011) as *r*^2^. Each “dot” represents the mean LD for that set of binned SNP-pairs. The color of the dot illustrates the number of SNP-pairs contributing to the mean; the color scale is shown in the right vertical bar. The blue line is a loess regression line of best fit, and the purple line corresponds to *r*^2^_max_/2. (A) Masindi, MS; (B) Namutumba, NB; and (C) Otuboi, OT.

The mean bin-wise LD in *Gff* has a maximum value at *r*^2^_max_ = 0.4018, and decays with physical distance to reach half of this maximum value (*r*^2^_max_/2 = 0.2009) near 708 bp (Figure S3). When considering individual populations with smaller sample size, *r*^2^_max_ increases (Figure 3), and *r*^2^ reaches values between 0.5 and 0.6. The analyses of individual populations also show variation in the number of base pairs at which LD decayed by half (*r*^2^_max_/2): NB = 1359 bp, OT = 2334 bp, and MS = 2429 bp; Figure 3). KG was excluded from the analysis due to low sample size.

After applying an LD filter based on a Beta probability distribution to identify potential LD outliers, we obtained 24,372 out of 6,454,294 SNP-pairs (0.38%) with corrected *P* values ≤ 0.01 (see *Materials and Methods*). Rather than looking at each of these individual genomic locations, we then used this information to sort potential SNPs of interest from the genome screen conducted in BayeScan, as relevant SNPs might have not been identified as outliers in this analysis due to the lack of statistical power consequence of the small sample size (see following section).

### Selection analyses

#### Susceptibility to infection:

Table S1 shows the *Trypanosoma* spp. infection status of the flies from the four *Gff* collection sites included in this study. BayeScan analysis (Fischer *et al.* 2011) of infected and uninfected individuals did not detect any *F*_st_ outlier loci when all locations were grouped together (Figure S4). Additional LD filtering of the top 10% BayeScan alpha values from this comparison, followed by a genome scan for genes located within 1000 bp of any SNP in the pair, identified a total of 340 genes (136 with functional annotations; File S3). Among the most prevalent biological functions of these genes were: *trans*-membrane transport, transcription regulation, rRNA processing, DNA-replication, and metabolic processing. In the molecular function (MF) domain, some of the frequently occurring motifs were related to zinc ion binding, DNA binding, transferase activity, and oxidoreductase activity.

**Figure 4:**
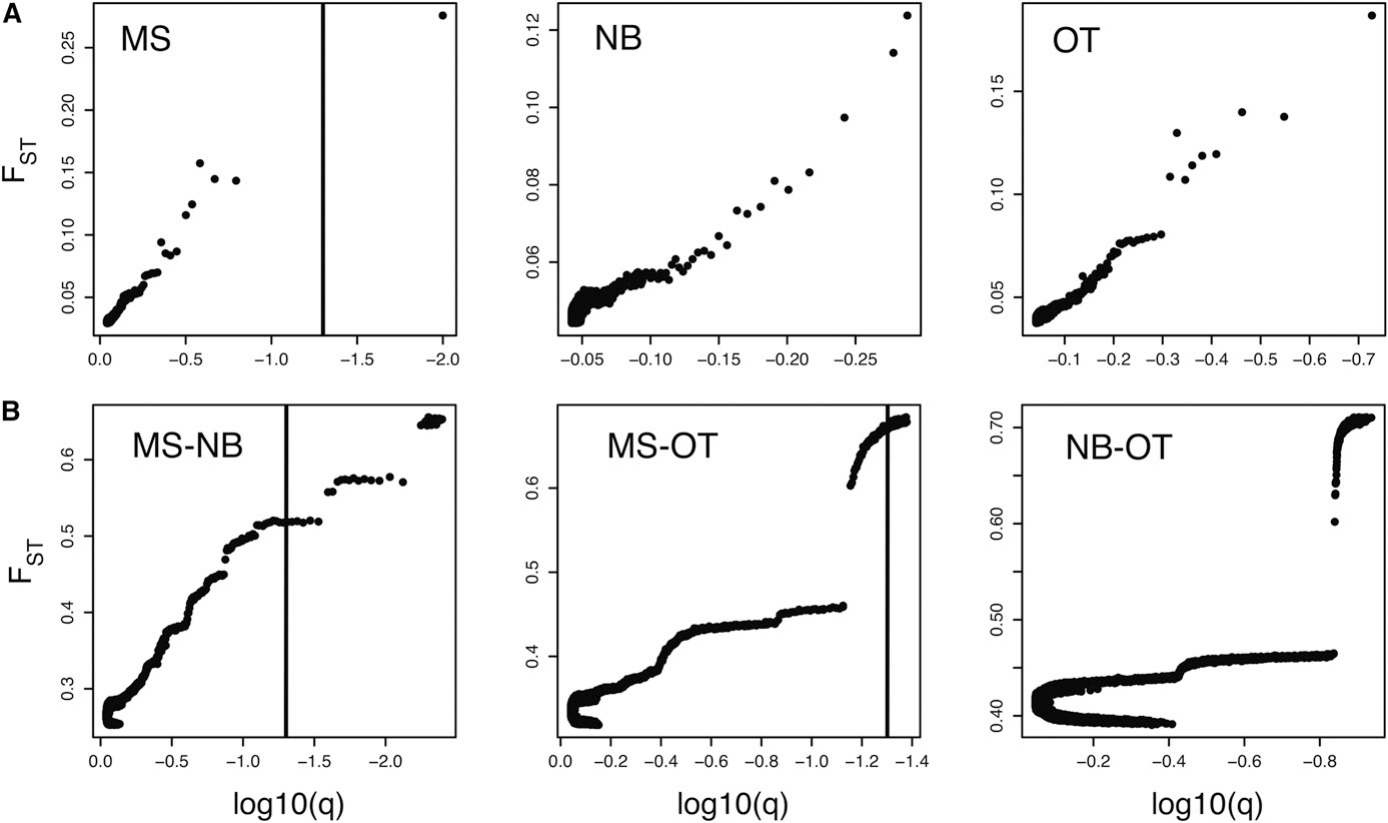
Bayescan *F*_st_ posterior probability plots. A screen for *F*_st_ outlier loci was performed on (A) each of three *Glossina fuscipes fuscipes* (*Gff*) populations from Uganda included in this study (both infected and uninfected flies), to identify SNPs associated with susceptibility to infection by Trypanosome; and (B) pairwise population comparisons, to identify SNPs associated with environmental local adaptation. The vertical line corresponds with the false discovery rate tradeoff value (FDR = 0.05) used to call outliers in BayeScan (Fischer *et al.* 2011). The population or pair of populations analyzed in each plot is indicated by a legend at the top left corner. MS, Masindi; OT, Otuboi; NB, Namutumba. Note that the KG (Kalangala Island) population was not included in this analysis due to an insufficient number of individuals.

When each location was analyzed individually (Figure 4A), we found one outlier locus in the MS dataset, located at JFJR01006593.1: 85294. No annotated genes were identified within a 1000-bp window of this SNP. We explored these results further by analyzing SNPs ranked within the top 5% and 10% alpha cutoff and that were common among each individual population analysis (Table S3). We identified 10 and 43 SNPs at the 5% and 10% alpha cutoff, respectively, and carried out a screen for putative genes under selection based on proximity, using a window of 1000 bp. This screen identified 20 genes, including transcription factors, and genes associated with circadian rhythms and membrane integrity. The genes and their distance to each SNP are found in File S3.

#### Local environmental adaptation:

We searched for SNPs associated with local environmental adaptation by searching for *F*_st_ outlier loci in pairwise comparisons contrasting samples from the three distinct genetic clusters from northern, southern, and western Uganda, as these populations experience different environmental regimes that differ mainly in precipitation and seasonality (File S1). Using BayeScan we did not identify any SNPs between NB and OT, but found 40 outlier loci between MS and NB, and 119 outlier loci between MS and OT (Figure 4B; File S4). Two of these outliers were common to both pairwise comparisons (Scaffold150:13771 and Scaffold150:13772; File S3) and are adjacent to each other, falling within a 100 bp window that includes three SNPs, and has a Tajima’s *D* value of 1.25 (Table S2). A genomic scan around these SNPs identified genes GFUI009284 and GFUI009279 within a region 1000 bp; unfortunately, no functional annotations were available for any of these genes (File S3). Eight of the 40 outlier loci between MS and NB also passed the LD filter, with two of these SNPs located within genes, and one located 405 bp apart, none of them with functional annotations (File S3). Of the 119 outlier SNPs from the MS *vs.* OT comparison, 60 had LD levels above the threshold, with 35 genes located within 1000 bp of them (File S3). We could not carry out this analysis for the NB *vs.* OT comparison because no outliers were detected by the BayeScan method.

PCadapt (Duforet-Frebourg *et al.* 2014) was used as an independent method to identify SNPs specifically associated to local adaptation. This analysis identified six SNPs as contributors to the two main factors discriminating among three genetic clusters (Table S4 and Figure S5), clusters that are consistent with the ones detected by the fastStructure and the PCA analyses (Figure 2), and match the geographical location of the sampling sites (Figure 1). Interestingly, two of these SNPs (Scaffold150: 13772 and 13773) had nearby annotated genes that were also identified using BayeScan: GFUI009284 and GFUI009279 (File S3). Only one of the six SNPs identified by PCadapt was part of an SNP-pair that passed the LD filter (Scaffold368: 310507), but no genes were located within 1000 bp of either SNP of the LD pair.

The haplotype-based analysis conducted in Hapflk (Fariello *et al.* 2013) on all populations, with *K* = 3 *a priori* number of clusters to reflect the population structure of the dataset (Figure 2), did not find any region with significant signatures of selection (Figure S6).

## Discussion

### Marker development, genetic diversity, and LD

Using a combination of ddRAD sequencing and WGS, we identified 73,297 variable sites across the genome of *Glossina fuscipes fuscipes* flies from Uganda. Advantages of using ddRADs include that they are randomly distributed across the genome, and that they are affordable to obtain enough read coverage to detect polymorphic loci even when at lower frequencies because only a fraction of the genome is being sequenced. Due to the low quality of six of the original NB samples, we combined the ddRAD dataset with WGS data, achieving higher sequence coverage for our original (low quality) samples while adding data from 10 additional individuals of the NB population to the dataset. The depth of coverage achieved by our method (∼40 ×) provides us with a high probability of observing both alleles of an individual, if in a heterozygote state, assuming random detection of each allele. The use of two restriction enzymes during the ddRAD library preparation, in contrast to similar techniques (Miller *et al.* 2007; Baird *et al.* 2008) that randomly sheared the genome, provides consistency in marker recovery increasing the chances that the same loci will be sequenced across all individuals, thus reducing the amount of missing data.

We identified an average of two SNPs for every 10,000 bp of the *Gff* genome covered, and used them to estimate LD in this species. This information is needed because associations between genes and phenotypic traits depend upon the existence of nonrandom associations between linked loci to identify causative genetic variants. This is the first study to estimate LD across the *Gff* genome. In fact, it is the first study to provide LD estimates from any *Glossina* species. LD decay needs to be considered when designing GWAS, as it affects the number of markers and samples required to achieve the power needed to identify the genetic basis of phenotypic variants. Organisms with rapid LD decay will require many more markers to detect associations, while those with slow LD decay will require less markers but provide less resolution for mapping the associations. The theoretical maximum value that LD measured as *r*^2^ can reach is *r*^2^_max_ = 0.43051 due to its dependence on allele frequencies (VanLiere and Rosenberg 2008); the LD estimated across three *Gff* populations is consistent with this expectation (Figure S3). Average LD in *Gff* decays in half (*r*^2^_max_ / 2 = 0.2009) at a distance of 708 bp. In comparison, *Drosophila melanogaster* achieves this *r*^2^ value (0.2) at 10 bp in autosomes, and 30 bp in the X chromosome (Mackay *et al.* 2012), *Anopheles arabiensis* at 200 bp (Marsden *et al.* 2014), and honeybee at 500 bp (Wallberg *et al.* 2014). The average size of LD blocks in *Anopheles gambiae* are estimated to be ∼40 bp (Wang *et al.* 2015).

The higher LD observed in *Gff* might be a consequence of a smaller effective population size (N_e_), and higher level of genetic structuring compared to either *Anopheles* or *Drosophila* populations. The average genome-wide genetic diversity (π) of the NB population, calculated from WGS data, is consistent with a small population size. The value of π for this population is one or two orders of magnitude lower (π = 0.00056) than reported values for *Drosophila* (0.0047–0.0114: Ometto *et al.* 2005; Fabian *et al.* 2012; Mackay *et al.* 2012), *Anopheles gambiae* (0.0043–0.0208: Stump *et al.* 2005; Chang *et al.* 2012), or *An. Arabiensis* (0.0020–0.0064: Stump *et al.* 2005; Cohuet *et al.* 2008; Marsden *et al.* 2014). Likewise, if one considers only synonymous sites, the value of π = 0.0001, compared to the corresponding value estimated in *Drosophila* of π = 0.0112 (Mackay *et al.* 2012).

A relatively small population size of *Gff* populations has been also previously reported in the literature. Estimated values of N_e_ for Ugandan *Gff* populations range from 33 to 310 when estimated from microsatellites (Hyseni *et al.* 2012), and fall below 500 individuals when estimated from mitochondrial data (Beadell *et al.* 2010). In contrast, N_e_ values estimated for *An. arabiensis* and *An. gambiae* range among thousands of individuals (Taylor *et al.* 1993; Hodges *et al.* 2013), and *Drosophila* populations in Africa have been estimated to have a N_e_ of over a million (Charlesworth 2009). Likewise, *Gff* genetic clusters are geographically structured at the local scale (Figure 1 and Figure 2; Beadell *et al.* 2010; Echodu *et al.* 2011; Hyseni *et al.* 2012; Aksoy *et al.* 2013), while fine geographic structure is not evident in African populations of *Anopheles* (Lanzaro *et al.* 1998; Moreno *et al.* 2007), and *Drosophila* (Hamlin *et al.*and Veuille 1999; Dieringer *et al.* 2005; Pool and Aquadro 2006; Pool *et al.* 2012).

Given the slow LD decay found in this study for wild *Gff* populations, the number of markers required to perform GWAS may need to be modified depending on the strength of selection acting on the trait of interest. Traits that are under strong selection, like those related to local environmental adaptation, may generate larger LD regions and thus might require less markers than those under a weaker selection regime, or those involving complex genotypic interactions, like susceptibility to trypanosome infection. For example, LD measured within a region of 57 kb around the *Drosophila delta* gene, which affects bristle variation and is under selection, found values of *r*^2^ of 0.3 to extend up to 5 kb (Long *et al.* 1998). LD blocks around the *para* gene, which provides insecticide resistance in *An. arabiensis*, extended for 2.4 Mb (Wang *et al.* 2015).

Another factor to take into account while selecting markers for GWAS is the fact that different populations of the same species may differ in their degree of genomic LD depending on their age and demographic histories. Populations established from a small number of founders after a bottleneck would have longer tracks of LD relative to those established from a large number of individuals (Reich *et al.* 2001). Conversely, older populations are likely to have shorter LD tracks because the genome has experienced recombination for a longer period of time. When LD was measured for the individual *Gff* populations, we found an *r^2^*_max_ value larger than that obtained from all the individuals combined (*r^2^*_max_ = 0.5–0.6 *vs.* 0.4). This may be explained if the smaller sample size of the individual populations results in the estimation of more homogeneous allele frequencies. The data also shows that LD decayed faster in NB than in OT and MS (Figure 3), with MS having the slowest rate of genome LD decay overall reaching half of *r*^2^_max_ at 2429 bp (compared to *r*^2^_max_ / 2 = 1359 bp in NB and 2334 bp in OT). A plausible explanation for this pattern could be that MS has undergone a recent bottleneck. This is consistent with the bottleneck evidence found by Beadell *et al.* (2010) and Echodu *et al.* (2011) using microsatellite data. Microsatellite data also points to some evidence of a bottleneck in OT, but the data are not conclusive (Echodu *et al.* 2011). The shorter LD tracks observed in NB could also be explained by the age of the population. Using genetic assignment tests on microsatellites it has been inferred that *Gff* in Uganda migrates from the southeast to the northwest (Aksoy *et al.* 2013), which would be consistent with NB being the oldest population from the group. Interpopulation differences in genomic LD were obscured when we estimated LD from all populations combined, and could explain why genetic associations were more likely to be identified when the analyzed datasets included the MS population, which had the strongest LD from the populations analyzed.

### Population structure

Genetic clustering of the four Ugandan *Gff* populations is based on geography, rather than on infection state (Figure 2A). Clustering analysis on the SNP dataset groups the four Ugandan populations in three distinct genetic clusters (Figure 2, A–C), and suggests that MS, NB, and KG are less differentiated from each other than to OT, consistent with results from mitochondrial and microsatellite data (Beadell *et al.* 2010; Hyseni *et al.* 2012; Echodu *et al.* 2013). Pairwise *F*_st_ values calculated from microsatellites (Echodu *et al.* 2013) suggest a similar level of genetic differentiation between OT, NB, and MS, while values estimated from SNPs suggest that OT is more genetically differentiated from NB than it is from MS. The SNP data also suggest that MS is closer to NB than it is to OT.

Subsequent work expanding the sample size and spatial breath of Ugandan *Gff* collections to accurately capture the SNP diversity across the species range will allow the identification of unique SNPs signatures that can subsequently be used to develop low cost genotyping tools to efficiently identify population of origin. This will provide insights into the difference between patterns of genetic differentiation found between the SNP and microsatellite/mtDNA datasets—differences that are currently based on the comparison with the SNP dataset of only four populations.

### Regions under selection

The *Gff* genome is estimated to have ∼20,749 genes (VectorBase 2014: *Glossina fuscipes* Gfusl1 assembly; Giraldo-Calderon *et al.* 2015). Using our SNP dataset, we performed a genomic screen to search for genes that may impact the vector competence of flies to trypanosome infection and to local environmental adaptation.

With our current individual sample size we identified one locus in the MS population with signatures of selection in relation to the infection status of the flies (Figure 4A). The complexity of the parameters that determined the infection state of a fly might have complicated detection of loci associated with this trait, especially at this low sample size. However, the power of detection is expected to significantly increase with the number of individuals genotyped. In humans, who have a genome 6 × larger than that of *Gff*, the slope of the power curve plateaus when the number of individuals reaches the thousands when dealing with genes with large magnitude effects and using ∼300–500K markers (Klein 2007). The percentage of trypanosome-infected flies varies widely across populations in Uganda (0–40%; Alam *et al.* 2012). In order to have significant power to detect additional loci under selection for this phenotype we need to screen many more samples.

Another caveat is the effect of age of the fly at the moment of capture, as younger flies are less likely to have ingested an infected blood meal compared to older flies and thus might not have been exposed to the parasite yet. Although all the flies included in this study were adults, we did not control for age. Future studies should account for these parameters in order to increase the probability of identifying genetic variants related to susceptibility of infection by trypanosomes. Furthermore, establishing an accurate diagnostic assay for the specific trypanosome species infecting the flies (*i.e.*, infections with HAT *vs.* AAT causing parasites), would both increase the power of the analysis and provide specific information on human disease risk.

The second genomic screen seeking loci involved in local environmental adaptation identified over 150 SNPs associated with geographic location. Using two independent methods, pairwise-population comparison in BayeScan (Fischer *et al.* 2011; Figure 4B) and PCadapt (Duforet-Frebourg *et al.* 2014; \ure S5), we identified regions under selection between populations in the west (MS), and east (NB and OT) of Uganda. Of particular interest is the genomic region around Scaffold150, positions 13771–13773, where three SNPs were identified as potential targets of selection. These SNPs are proximate to genes GFUI009284 and GFUI009279, which unfortunately lack homologs or a functional annotation (File S3, File S4, and Table S4).

Analysis of the bioclimatic parameters for each of the populations (Hijmans *et al.* 2005; File S1) indicates that the seasonality of precipitation as well as temperature and precipitation during the wettest month of the year are different in MS compared to the eastern populations (NB and OT). Tsetse flies are associated with riverine habitats and vegetation thickets along rivers, which they use to get relief from heat and desiccation and to seek hosts upon which to feed (Dyer *et al.* 2011). Given their life history, and that they are extremely sensitive to temperature and precipitation (Nash 1933; Hargrove 2001a, 2001b; Terblanche *et al.* 2008), we can hypothesize that the genomic region identified as relevant to local environmental adaptation might, for example, regulate the ability to resist desiccation.

### Epidemiological relevance and future research

*Gff* flies cause major public health concern and economic losses in Uganda due to the pathogenic parasites they transmit. We have developed a set of 73,297 genotyping markers across the genome of *Gff*, provided information on LD patterns, conducted a preliminary study of the pattern of genetic differentiation revealed by these markers, and performed a pilot study looking for gene regions under selection using a variety of methods that account for drift and population structure, and incorporate the information from the LD analyses.

Specifically, we searched for genomic regions responsible for tsetse’s resistance/susceptibility phenotype to trypanosome infections and to different environmental adaptations. Identifying genetic regions associated with Trypanosoma infections could inform about (a) the genetic basis for resistance to trypanosomes in natural *Gff* populations, and (b) if different *Gff* genotypes vary in their transmission ability of *Tbg* and *Tbr* parasite species. This in turn could provide an immediate mechanism of action against the spread of the disease via the introduction of refractory genes into wild populations. Knowledge of the environmental parameters involved in generating and maintaining the genetic differences among *Gff* populations will contribute to develop more realistic suitability maps for this vector than currently available, as the integration of the ecological and evolutionary axes of divergence will likely increase their predictive power, and thus our ability to forecast changes in the vector distribution in response to impeding change in climatic conditions. The results of this study demonstrate the efficacy of our approach, and provide baseline data for future work to look at the genetic underpinning of these epidemiologically important traits.

## Supplementary Material

Supplemental Material
