# Supplementary material for: Patterns of Genome-Wide Variation in Glossina fuscipes fuscipes Tsetse Flies from Uganda
Source: G3 (Bethesda). 2016 Mar 26;6(6):1573–84. doi: 10.1534/g3.116.027235 (PMC4889654; doi:10.1534/g3.116.027235)
Supplement: Supplemental Material [file supp_6_6_1573__index.html]

Supplemental Material 

# Patterns of Genome-Wide Variation in *Glossina fuscipes fuscipes* Tsetse Flies from Uganda

Supplemental Material for Gloria-Soria *et al.*, 2016

Supplemental Material

**Files in this Data Supplement:**

- Figure S1 - Manhattan plot of Tajimasï¿½D values computed from 73, 297 SNPs in 1000 bp windows across the *Glossina fuscipes fuscipes* reference supercontigs. (.pdf, 335 KB)
- Figure S2 - Genetic membership bar plot based on 73, 297 SNPs SNPs obtained using fastStructure (Raj et al. 2014). (.pdf, 73 KB)
- Figure S3 - Decay of linkage disequilibrium with physical distance in *Glossina fuscipes fuscipes*. (.pdf, 197 KB)
- Figure S4 - Bayescan Fst posterior probabilty plot on populations of four *Glossina fuscipes fuscipes* from Uganda (infected and uninfected flies) to identify SNPs associated with susceptibility to infection by Trypanosome. (.pdf, 67 KB)
- Figure S5 - Results from the PCadapt analysis (Duforet-Frebourg 2014) on the MS, KG and OT populations. (.pdf, 81 KB)
- Figure S6 - Hapflk was run with default parameters and *K*=3. (.pdf, 190 KB)
- File S1 - Bioclimatic information for the four *Gff* populations from Uganda used in this study (Hijmans et al. 2005). (.pdf, 161 KB)
- File S2 - Selection analysis. (.pdf, 217 KB)
- File S3 - Genes located within 1000 bp of SNPs identified as 1) outliers by BayeScan in pairwise population comparisons for local adaptation; 2) SNPs ranked within the BayeScan top 10% alpha values and common to all individual population comparisons for trypanosome infection status; and 3) SNPs ranked within the BayeScan top 10% alpha values, for trypanosome infection status when all populations were grouped together, filtered by LD. (.xls, 126 KB)
- File S4 - SNPs identified as outliers in BayeScan during the pairwise population comparison to detect environmental local adaptation candidates. (.xls, 18 KB)
- Table S1 - Detailed individual sample information (KG: Kalangala; MS: Masindi; OT: Otuboi; NB: Namutumba; Infection = Trypanosomes detected in sample; WGS= whole genome sequencing). (.txt, 2 KB)
- Table S2 - Tajima's D values measured across the *Gff* genome using the 73, 297 SNPs and a window size of 1000bp. Only windows with at least one SNP are listed. (.xls, 4044 KB)
- Table S3 - List of SNPs ranked within the top 10% BayeScan alpha values in the infection status analysis performed on all populations simultaneously (infected vs uninfected flies), and list of SNPs common to the 10% ranked alpha values across all individual population analyses for infection status. (.xls, 509 KB)
- Table S4 - Tentative candidates for local adaptation as determined by PCadapta (Duforet-Frebourg 2014). (.pdf, 39 KB)
