# Supplementary material for: Patterns of Genome-Wide Variation in Glossina fuscipes fuscipes Tsetse Flies from Uganda
Source: G3 (Bethesda). 2016 Mar 26;6(6):1573–84. doi: 10.1534/g3.116.027235 (PMC4889654; doi:10.1534/g3.116.027235)
Supplement: Supplemental Material [file supp_g3.116.027235_FigureS1.pdf]

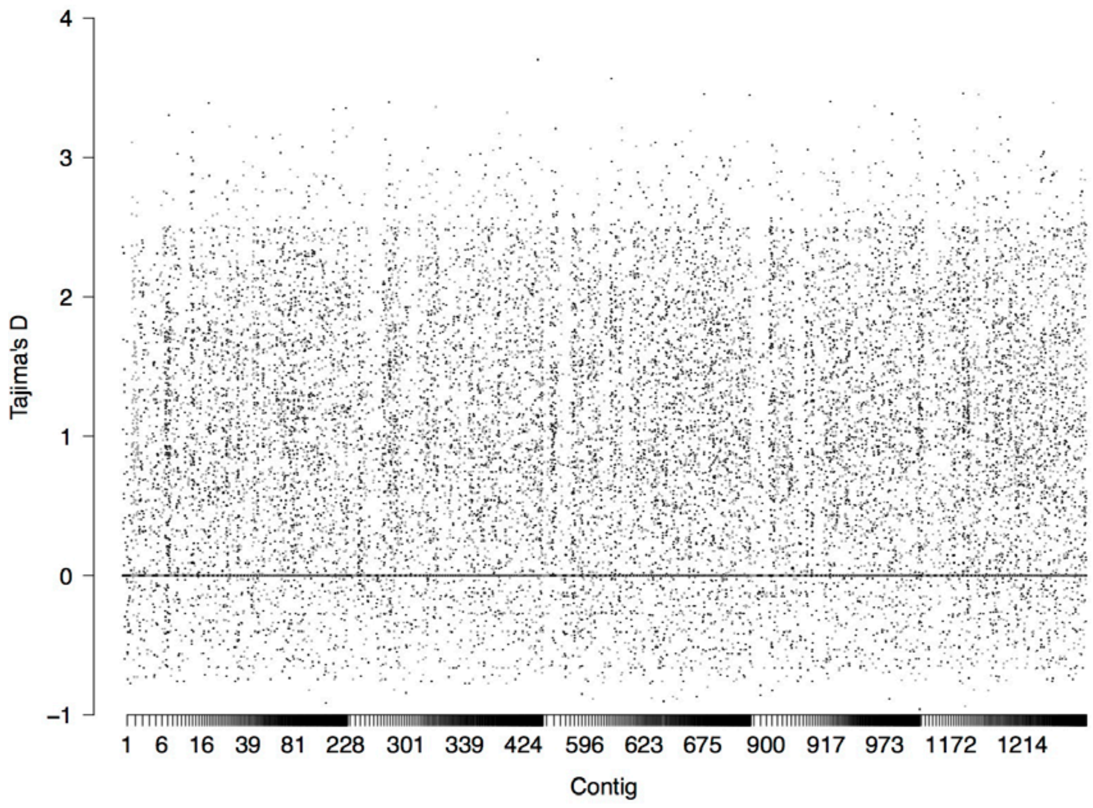

Figure S1: Manhattan plot of Tajimas'D values computed from 73, 297 SNPs in 1000 bp windows across the *Glossina fuscipes fuscipes* reference supercontigs. Only windows containing at least one SNP were considered. Mean Tajima's D = 0.4045. Contig order is arbitrary.
