# Supplementary material for: Patterns of Genome-Wide Variation in Glossina fuscipes fuscipes Tsetse Flies from Uganda
Source: G3 (Bethesda). 2016 Mar 26;6(6):1573–84. doi: 10.1534/g3.116.027235 (PMC4889654; doi:10.1534/g3.116.027235)
Supplement: Supplemental Material [file supp_g3.116.027235_FigureS2.pdf]

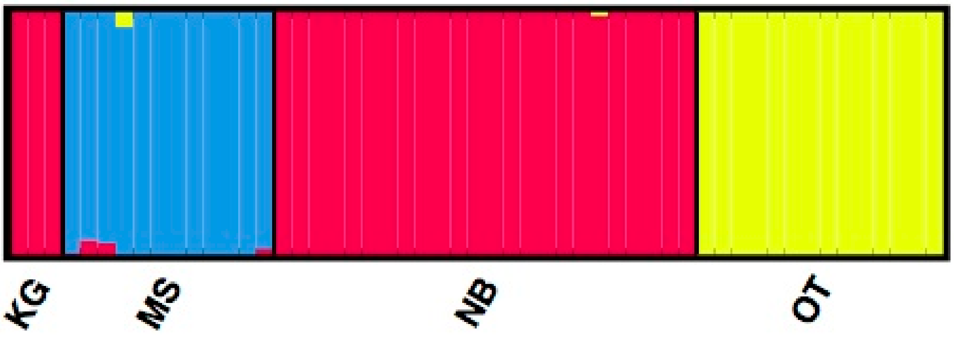

Figure S2: Genetic membership bar plot based on 73, 297 SNPs SNPs obtained using fastStructure (Raj et al. 2014). Each vertical bar represents a single individual. The height of each color represents the probability of assignment to each of *K= 3* clusters for all sampled flies in all populations (N=53). KG: Kalangala; MS: Masindi; OT: Otuboi; NB: Namutumba.
