# Supplementary material for: Patterns of Genome-Wide Variation in Glossina fuscipes fuscipes Tsetse Flies from Uganda
Source: G3 (Bethesda). 2016 Mar 26;6(6):1573–84. doi: 10.1534/g3.116.027235 (PMC4889654; doi:10.1534/g3.116.027235)
Supplement: Supplemental Material [file supp_g3.116.027235_FigureS3.pdf]

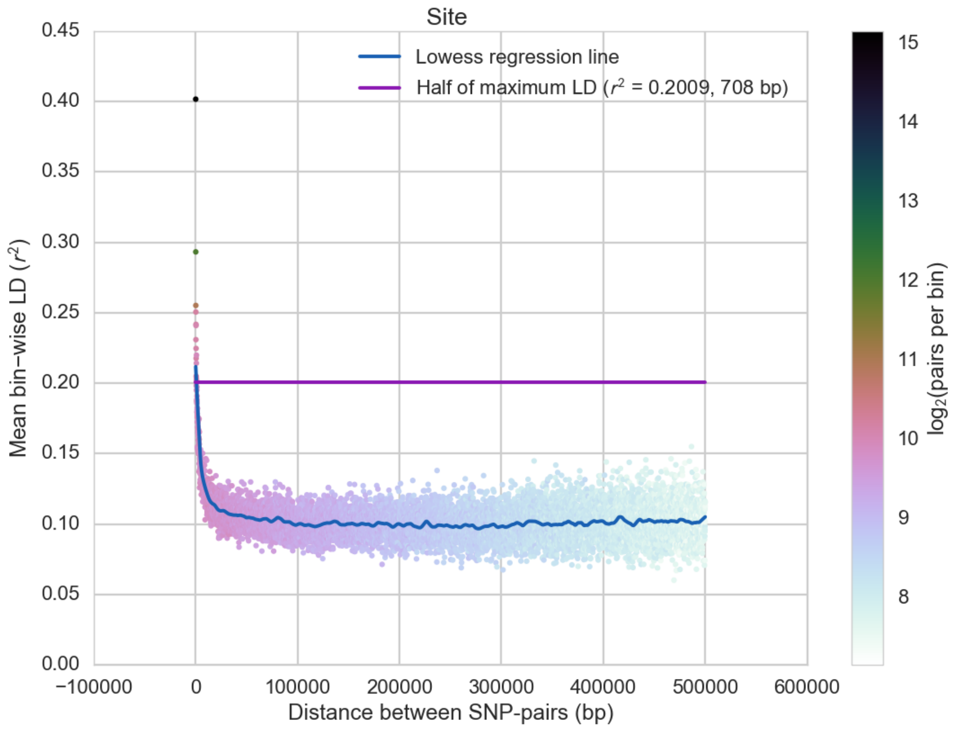

Figure S3: Decay of linkage disequilibrium with physical distance in *Glossina fuscipes fuscipes*. Pairwise LD between SNPs located in the same supercontig was estimated as r^2 from all individuals of all populations using Vcftools v. 0.1.12 (Danecek et al. 2011). Each point represents the mean LD for that set of binned SNP-pairs. The color of the point illustrates the number of SNP-pairs contributing to the mean; the color scale is shown in the right vertical bar. The blue line is a lowess regression line of best fit and the purple line indicates the value of $r^2_{max}/2$.
