# Supplementary material for: Patterns of Genome-Wide Variation in Glossina fuscipes fuscipes Tsetse Flies from Uganda
Source: G3 (Bethesda). 2016 Mar 26;6(6):1573–84. doi: 10.1534/g3.116.027235 (PMC4889654; doi:10.1534/g3.116.027235)
Supplement: Supplemental Material [file supp_g3.116.027235_FigureS4.pdf]

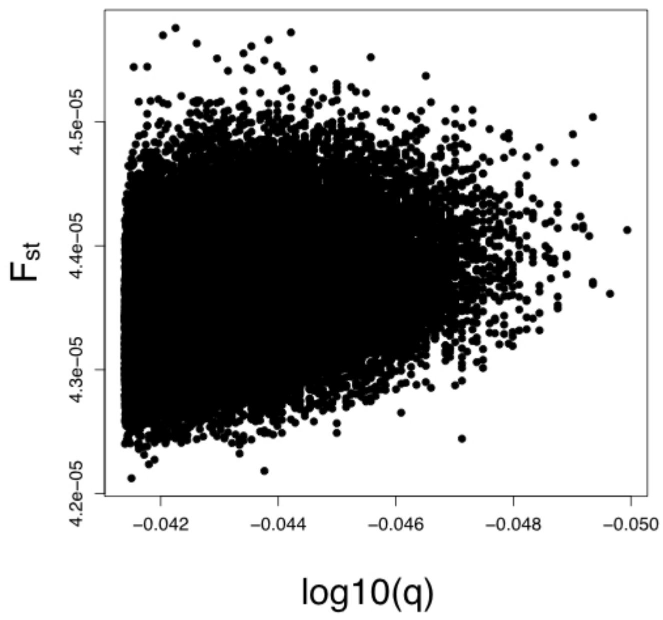

Figure S4: Bayescan F_{st} posterior probabilty plot on populations of four *Glossina fuscipes fuscipes* from Uganda (infected and uninfected flies) to identify SNPs associated with susceptibility to infection by Trypanosome. Analysis was performed on all individuals from the four populations together: Masindi (MS), Namutumba (NB), Otuboi (OT), and Kalangala Island (KG). Vertical line indicates the false discovery rate (FDR = 0.05) tradeoff used to call outliers in BayeScan (Fischer et al. 2011).
