# Supplementary material for: Patterns of Genome-Wide Variation in Glossina fuscipes fuscipes Tsetse Flies from Uganda
Source: G3 (Bethesda). 2016 Mar 26;6(6):1573–84. doi: 10.1534/g3.116.027235 (PMC4889654; doi:10.1534/g3.116.027235)
Supplement: Supplemental Material [file supp_g3.116.027235_FigureS5.pdf]

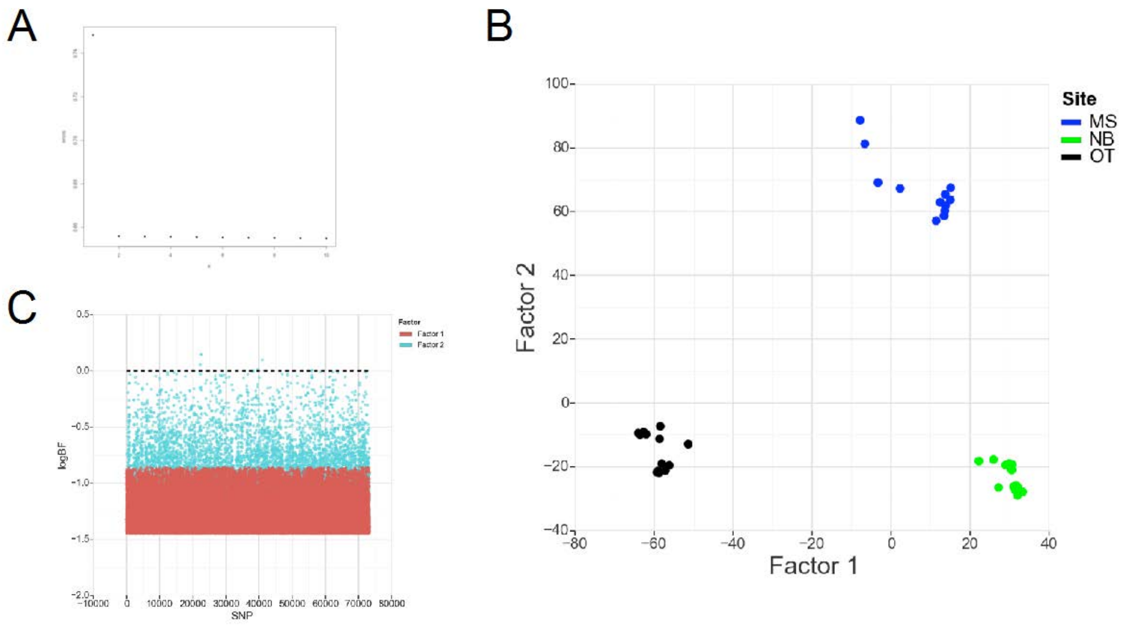

Figure S5: Results from the PCadapt analysis (Duforet-Frebourg 2014) on the MS, KG and OT populations. A) Selection of *K*=2.The Y axis is the mean squared error. The X-axis represents each run of PCAdapt with *K* ranging from 1 to 10 showing that values greater than 2 did not appreciably reduce the error. B) Tentative support for limited local adaptation. The Y-axis is the log10(Bayes Factor) score for each SNP. The X axis represents and arbitrary order of the SNPs and is not indicative of location along the genome. The larger the number on the Y axis, the more support there is for local adaptation. SNPs are colored by factor. The dotted line denotes a score generally interpreted as the minimum warranting a passing mention (Jeffreys 1998). C) PCAdapt factors group individuals into three distinct clusters.The X and Y axes represent the where each individual falls along the spectrum of latent factor 1 and 2, respectively. Each dot represents an individual fly. Individuals are colored by sampling site: MS (blue), NB (green), and OT (black).
