# Supplementary material for: Patterns of Genome-Wide Variation in Glossina fuscipes fuscipes Tsetse Flies from Uganda
Source: G3 (Bethesda). 2016 Mar 26;6(6):1573–84. doi: 10.1534/g3.116.027235 (PMC4889654; doi:10.1534/g3.116.027235)
Supplement: Supplemental Material [file supp_g3.116.027235_FigureS6.pdf]

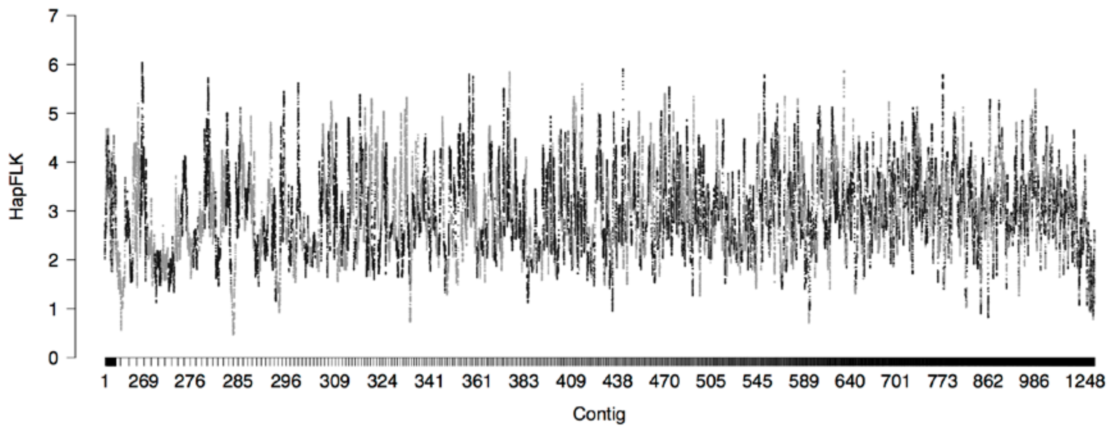

Figure S6*:* Hapflk was run with default parameters and *K*=3. Raw Hapflk values are plotted. Hapflk *p* values were obtained by fitting a linear model by robust regression using an M estimator as implemented in the R function "rlm" and adjusted for multiple tests using the Bonferroni method. After correction, no significant values were found. The order of the contigs is arbitrary.
