# Supplementary material for: Patterns of Genome-Wide Variation in Glossina fuscipes fuscipes Tsetse Flies from Uganda
Source: G3 (Bethesda). 2016 Mar 26;6(6):1573–84. doi: 10.1534/g3.116.027235 (PMC4889654; doi:10.1534/g3.116.027235)
Supplement: Supplemental Material [file supp_g3.116.027235_FileS1.pdf]

SELECTED BIOCLIMS

Site	bio12	bio13	bio14	bio15	bio18	bio19	bio2	bio3	bio6	bio7	bio4	bio5	bio8	bio9
KG	1918.0	302.0	79.0	45.0	436.0	306.0	103.0	83.0	153.0	124.0	525.0	277.0	219.0	208.0
OT	1312.0	194.0	17.0	51.0	134.0	447.0	129.0	79.0	165.0	163.0	1015.0	328.0	222.0	245.0
MS	1330.0	174.0	33.0	41.0	186.0	388.0	119.0	81.0	166.0	146.0	795.0	312.0	223.0	238.0
NB	1322.0	202.0	40.0	40.0	214.0	310.0	121.0	82.0	166.0	147.0	691.0	313.0	234.0	238.0

Table 1 Values of selected bioclims for each sampling location.

Temperature values are in degrees C * 10. Precipitation values are in mm.

bio2 = Mean Diurnal Range (Mean of monthly (max temp - min temp))
bio3 = Isothermality (BIO2/BIO7) (* 100)
bio4 = Temperature Seasonality (standard deviation *100)
bio5 = Max Temperature of Warmest Month
bio6 = Min Temperature of Coldest Month
bio7 = Temperature Annual Range (BIO5-BIO6)
bio8 = Mean Temperature of Wettest Quarter
bio9 = Mean Temperature of Driest Quarter
bio12 = Annual Precipitation
bio13 = Precipitation of Wettest Month
bio14 = Precipitation of Driest Month
bio15 = Precipitation Seasonality (Coefficient of Variation)
bio18 = Precipitation of Warmest Quarter
bio19 = Precipitation of Coldest Quarter

Copyright © 2016 Corresponding Author *et al.*
Manuscript compiled: Tuesday 5th January, 2016%

BIOCLIM DATA PCA RESULTS

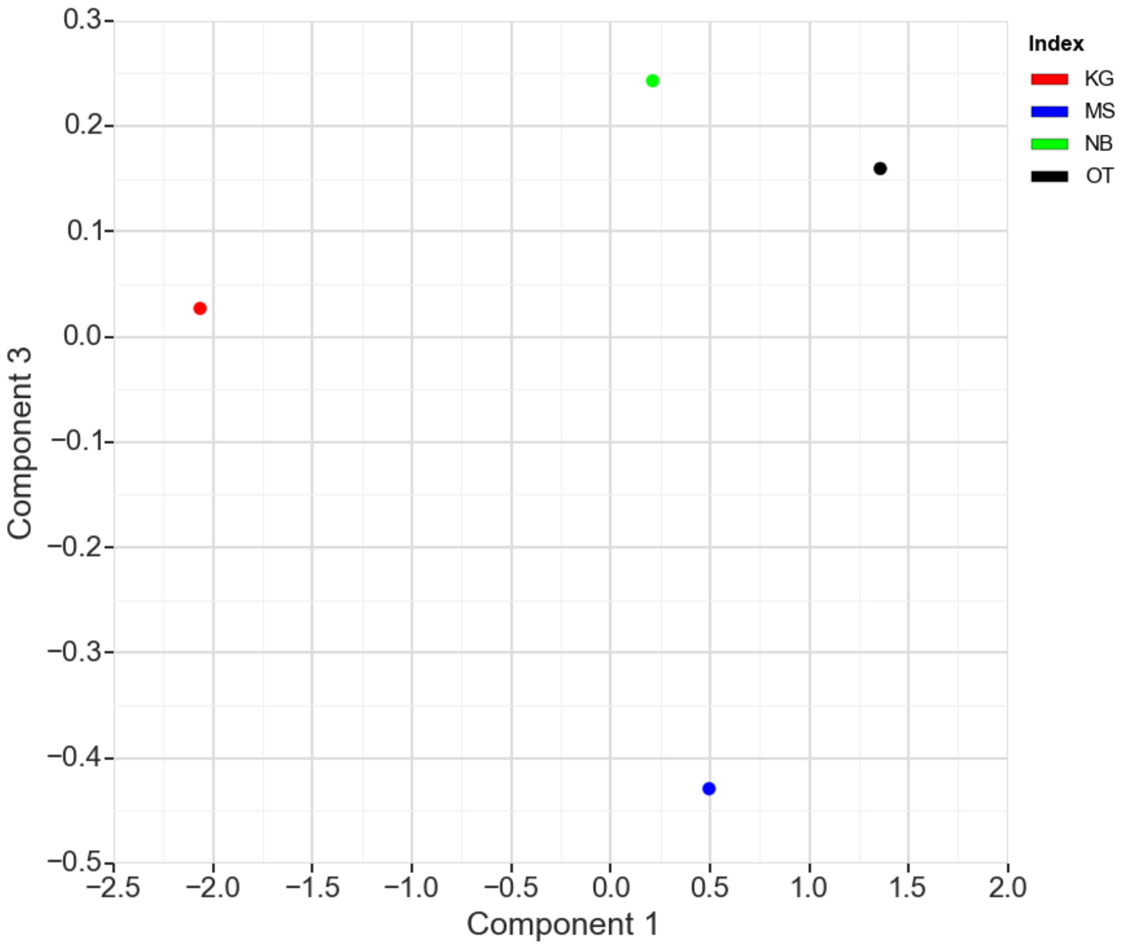

Figure 1 Principal components one and three. Principal components one and three. Components one and three plotted against each other illustrate that the MS site groups apart from the others along component three's axis. Investigation of component three's loading values reveals that the bioclims most responsible for this are bio8, bio15. and bio13.
