# Supplementary material for: Patterns of Genome-Wide Variation in Glossina fuscipes fuscipes Tsetse Flies from Uganda
Source: G3 (Bethesda). 2016 Mar 26;6(6):1573–84. doi: 10.1534/g3.116.027235 (PMC4889654; doi:10.1534/g3.116.027235)
Supplement: Supplemental Material [file supp_g3.116.027235_FileS2.pdf]

Selection analysis

The program BayeScan (Fischer et al. 2011) was used to perform a screen for F_{st} outlier loci, using a pairwise analysis between infected and uninfected flies and between population pairs experiencing different environmental conditions, at a False Discovery Rate of 0.05. The software assigns a posterior probability value to each loci based on the observed distribution of F_{st} under a model with and without selection. Outliers are those loci deviating from neutral expectations. BayeScan can perform analyses of a very small sample size like ours without introducing a particular bias (Foll 2012). However, as a small sample size might lack statistical power, we further identified potential candidate genes by searching for genes in the proximity of the SNPs with absolute alpha values ranked within the top 5%, and 10% (see next section). Alpha values generated by BayeScan indicate the strength and direction of selection, a positive value suggests diversifying selection, whereas a negative value suggests balancing or purifying selection (Beaumont and Balding 2004).

The dataset of SNPs ranked within the top 10% alpha values was subsequently filtered by LD to identify loci that were not designated as outliers by BayeScan, possibly due to insufficient statistical power, but that might be under selection as indicated by abnormal LD values. Those SNPs that were part of an SNP-pair with an assigned probability value of $p \leq 0.01$, based on a Beta probability distribution (see LD section in main text), were considered outliers.

We use the PCAdapt software (Duforet-Frebourg 2014) to independently identify SNPs that may be contributing to local environmental adaptation. The PCadapt program simultaneously identifies latent factors that allow the individuals to be partitioned into groups as well as SNPs that are atypically related to population structure as measured by the latent factors, and therefore may be located in regions involved in local environmental adaptation (Duforet-Frebourg 2014). The PCadapt method avoids possible artifacts generated by existing population subdivision (geographic differentiation), as it does not assume any specific model of population structure, providing a 2-fold or more reduction on the false discovery rate compared to BayesScan (Duforet-Frebourg 2014). The

analysis assigns Bayes Factor scores to each SNP and membership to one of the factor groups. Larger Bayes Factor scores indicate more support for local environmental adaptation. PCAdapt was run with a range of values of *K* from 1 to 10 using the VCF file containing individuals from sites MS, NB, and OT. After comparing the mean squared error for each of the runs, the run with *K*=2 was chosen because values greater than 2 did not appreciably reduce the error. SNPs that met or exceeded the threshold established using the Jeffreys' strength of evidence scale (Jeffreys 1998) were determined good candidates for local environmental adaptation.

Haplotype selection analysis was conducted with the hapFLK pipeline (Fariello et al. 2013) to detect signatures of selection based on reconstructed haplotypes. This method incorporates the hierarchical structure of the populations in the analysis, is robust to the demographic history of the populations, and is capable of detecting incomplete selective sweeps difficult to detect with methods that rely on allele frequencies (Fariello et al. 2013). We ran the software genome-wide with 3 *a priori* clusters, as determined by the FastStructure (Raj et al. 2014) analysis, and 20 runs of the expectation–maximization algorithm. HapFLK values with a $p<0.05$ were considered positive for selection, after correction to account for multiple tests by the Holm-Bonferroni method (Holm 1979).
