# Supplementary material for: Patterns of Genome-Wide Variation in Glossina fuscipes fuscipes Tsetse Flies from Uganda
Source: G3 (Bethesda). 2016 Mar 26;6(6):1573–84. doi: 10.1534/g3.116.027235 (PMC4889654; doi:10.1534/g3.116.027235)
Supplement: Supplemental Material [file supp_g3.116.027235_TableS4.pdf]

Table S4*:* Tentative candidates for local adaptation as determined by PCadapt[a] (Duforet-Frebourg 2014).

logBF	Scaffold	Location
0.145904	Scaffold150	13773
0.145718	Scaffold150	13772
0.098694	Scaffold250	396214
0.054760	Scaffold144	565217
0.012381	Scaffold207	475992
0.001190	Scaffold368	310507*

[a]Shown are the Contig locations for all SNPs assigned a logBF (log10(Bayes Factor)) scores of at least 0 by PCAdapt. This is considered a minimum significance level warranting mention.

*Indicates a SNP who is part of a SNP-pair that passed the LD filter.
